# Functional and Structural Insights Revealed by Molecular Dynamics Simulations of an Essential RNA Editing Ligase in *Trypanosoma brucei*

**DOI:** 10.1371/journal.pntd.0000068

**Published:** 2007-11-14

**Authors:** Rommie E. Amaro, Robert V. Swift, J. Andrew McCammon

**Affiliations:** 1 Department of Chemistry and Biochemistry, University of California San Diego, La Jolla, California, United States of America; 2 NSF Center for Theoretical Biological Physics (CTBP), University of California San Diego, La Jolla, California, United States of America; 3 Department of Pharmacology, University of California San Diego, La Jolla, California, United States of America; 4 Howard Hughes Medical Institute, University of California San Diego, La Jolla, California, United States of America; Swiss Tropical Institute, Switzerland

## Abstract

RNA editing ligase 1 (TbREL1) is required for the survival of both the insect and bloodstream forms of *Trypanosoma brucei*, the parasite responsible for the devastating tropical disease African sleeping sickness. The type of RNA editing that TbREL1 is involved in is unique to the trypanosomes, and no close human homolog is known to exist. In addition, the high-resolution crystal structure revealed several unique features of the active site, making this enzyme a promising target for structure-based drug design. In this work, two 20 ns atomistic molecular dynamics (MD) simulations are employed to investigate the dynamics of TbREL1, both with and without the ATP substrate present. The flexibility of the active site, dynamics of conserved residues and crystallized water molecules, and the interactions between TbREL1 and the ATP substrate are investigated and discussed in the context of TbREL1's function. Differences in local and global motion upon ATP binding suggest that two peripheral loops, unique to the trypanosomes, may be involved in interdomain signaling events. Notably, a significant structural rearrangement of the enzyme's active site occurs during the apo simulations, opening an additional cavity adjacent to the ATP binding site that could be exploited in the development of effective inhibitors directed against this protozoan parasite. Finally, ensemble averaged electrostatics calculations over the MD simulations reveal a novel putative RNA binding site, a discovery that has previously eluded scientists. Ultimately, we use the insights gained through the MD simulations to make several predictions and recommendations, which we anticipate will help direct future experimental studies and structure-based drug discovery efforts against this vital enzyme.

## Author Summary

RNA editing ligase 1 (TbREL1) is required for the survival of both the insect and bloodstream forms of *Trypanosoma brucei*, the parasite responsible for the devastating tropical disease African sleeping sickness. The type of RNA editing that TbREL1 is involved in is unique to the trypanosomes, and no close human homolog is known to exist. Here we use molecular dynamics simulations to investigate the dynamics of TbREL1, both with and without the ATP substrate present. The flexibility of the active site, dynamics of conserved residues and crystallized water molecules, and the interactions between TbREL1 and the ATP substrate are investigated and discussed. During the apo simulations, a significant structural rearrangement of the enzyme's active site opens an additional cavity adjacent to the ATP binding site that could be exploited in the development of effective inhibitors against this protozoan parasite. State-of-the-art electrostatics calculations reveal a novel putative RNA binding site, a discovery that has previously eluded scientists. Ultimately, we use the insights gained through the MD simulations to make several predictions, which we anticipate will help direct future experimental studies and structure-based drug discovery efforts against this vital enzyme.

## Introduction

The existence and widespread occurrence of several devastating trypanosomal tropical diseases, such as Chagas' disease and African sleeping sickness, cause an estimated 1 million deaths each year in developing countries [1]. In 2005, the completely sequenced genomes of *Trypanosoma brucei*, the causative agent of African sleeping sickness, *T. cruzi*, the causative agent of Chagas disease, and *Leishmania major*, the causative agent of Leishmaniasis, were published, yet, despite these great genomic successes, the need for effective and suitable drugs still remains [2]. Currently available drugs were developed in the first half of the twentieth century and they are toxic, difficult to deliver and often ineffective [3].

The trypanosome pathogens responsible for these diseases all share unique post-transcriptional mRNA editing features, the discovery of which revealed a rich addition to the central dogma of biology, in which information not only passes from DNA to RNA to protein, but also between different classes of RNA [4]. Through the insertion and deletion of uridylates (U's), the editing process transforms premature mitochondrial RNA (pre-mRNA) to mature mRNA in a multi-protein complex known as the editosome [5],[6]. The exact composition of the editosome complex has yet to be fully characterized, although 20S core complexes have a M_w_ of 1.6 MDa and appear to be comprised of 16-20 proteins [7]. To further complicate matters, it has recently been demonstrated that at least three different 20S editosomes of heterogeneous composition and distinct specificity are involved in the editing process [8], possibly reflecting compositional changes of this dynamic multicatalyst complex at different stages in the editing process [5].

**Figure 1:**
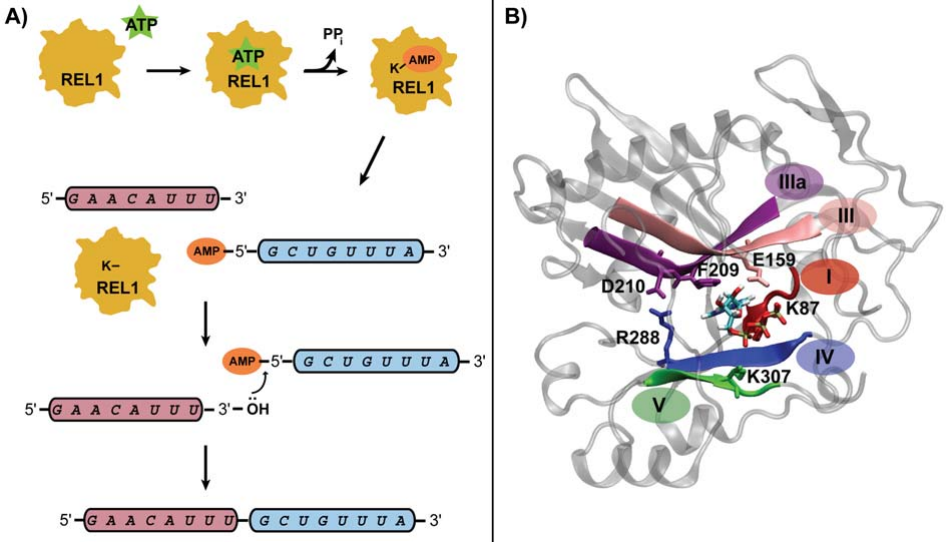
RNA editing ligase 1 reaction and structure. A) The three-step ligation reaction catalyzed by RNA editing ligase 1. B) The energy minimized TbREL1 structure is depicted with ATP-bound in the active site. The crystallographic water molecules and explicit solvent are included in the simulations, but omitted in the figure for clarity. The five conserved ligase motifs are colored and labeled, and key conserved residues within each motif are depicted in licorice.

The remarkable mRNA editing process begins in the trypanosomal mitochondrial (mt) DNA, which consists of a topologically linked network of thousands of minicircles and dozens of maxicircles. It is the transcripts of these maxicircles, encoding components of respiratory complexes and energy transduction systems, which undergo extensive RNA editing. The editing process begins when guide RNAs (gRNAs) are transcribed from the minicircles in the mt genome and subsequently base-pair with pre-mRNA sequences through a conserved “anchor sequence” [9],[10]. Endonucleolytic cleavage of the pre-mRNA strand occurs at a point of mismatch between the trans-acting gRNA and its cognate pre-mRNA, by endonucleolytic enzymes that have yet to be characterized. Depending on the type of RNA mismatch, U's are either added, by terminal uridylyl transferase (TUTase), or deleted, by a U-specific 3′ exonuclease (ExoUase). The processed RNA fragments are then religated by one of two RNA ligases, Kinetoplastid RNA editing ligase 1 (TbREL1) or RNA editing ligase 2 (TbREL2), generally depending on whether the process is deletion or insertion editing, respectively. Religation of the now completely base-paired double stranded RNA (dsRNA) strands occurs in a three-step process (Fig. 1A). In the first step, the catalytic lysine residue acts in concert with a divalent metal cofactor within the ATP binding pocket of TbREL1 and is autoadenylated, forming a protein-AMP intermediate and releasing pyrophosphate. In the next step, a 5′-5′ phosphate linkage is formed when the AMP is transferred to the 5′ end of the nicked 3′ RNA substrate, which is proximal to the active site. Finally, the ligation process is completed when the 3′ hydroxyl group of the other strand displaces the 5′ AMP, resulting in a new phosphodiester bond.

TbREL1 has been shown to play a key role in the vitality of *Trypanosoma brucei*, as it is required for survival of both the insect and bloodstream forms of the pathogen [11],[12]. TbREL1 is comprised of a catalytic N-terminal adenylation domain and a C-terminal domain that facilitates non-covalent interaction with another editosome protein, KREPA2 [13]. Interestingly, an oligonucleotide binding (OB-fold) domain, usually associated with DNA ligases and capping enzymes *in cis*, appears to be provided by KREPA2 *in trans* and has been predicted to act as a conformational switch regulating various steps in the editing process [13]. The recently crystallized 1.2 Å resolution structure of the adenylation domain, TbREL1, from *T. brucei* with the bound ATP ligand revealed a number of interesting features of the enzyme, including an unusual water-solvated ATP binding pocket [14]. As the N-terminal domain alone has shown to be capable of RNA ligation activity, further studies characterizing its functional dynamics are relevant. Interestingly, although a single Mg^2+^ ion is clearly coordinated to the protein and ATP in the crystal structure, the protein is not yet adenylated, indicating that the structure is in a pre-catalytic conformation. Furthermore, it has been suggested for this system, and shown experimentally for related systems, that the nucleotidyl transfer reaction proceeds by a two Mg^2+^ mechanism [15]. It has been hypothesized that the first Mg^2+^ binds with high affinity between the ATP β− and γ−phosphate groups, as seen in the TbREL1 crystal structure, and the second magnesium coordinates to a lower-affinity site between the ATP α-phosphate group and catalytic lysine, subsequently promoting the bond cleavage between the α and β phosphate and the adenylyl transfer [14].

**Figure 2:**
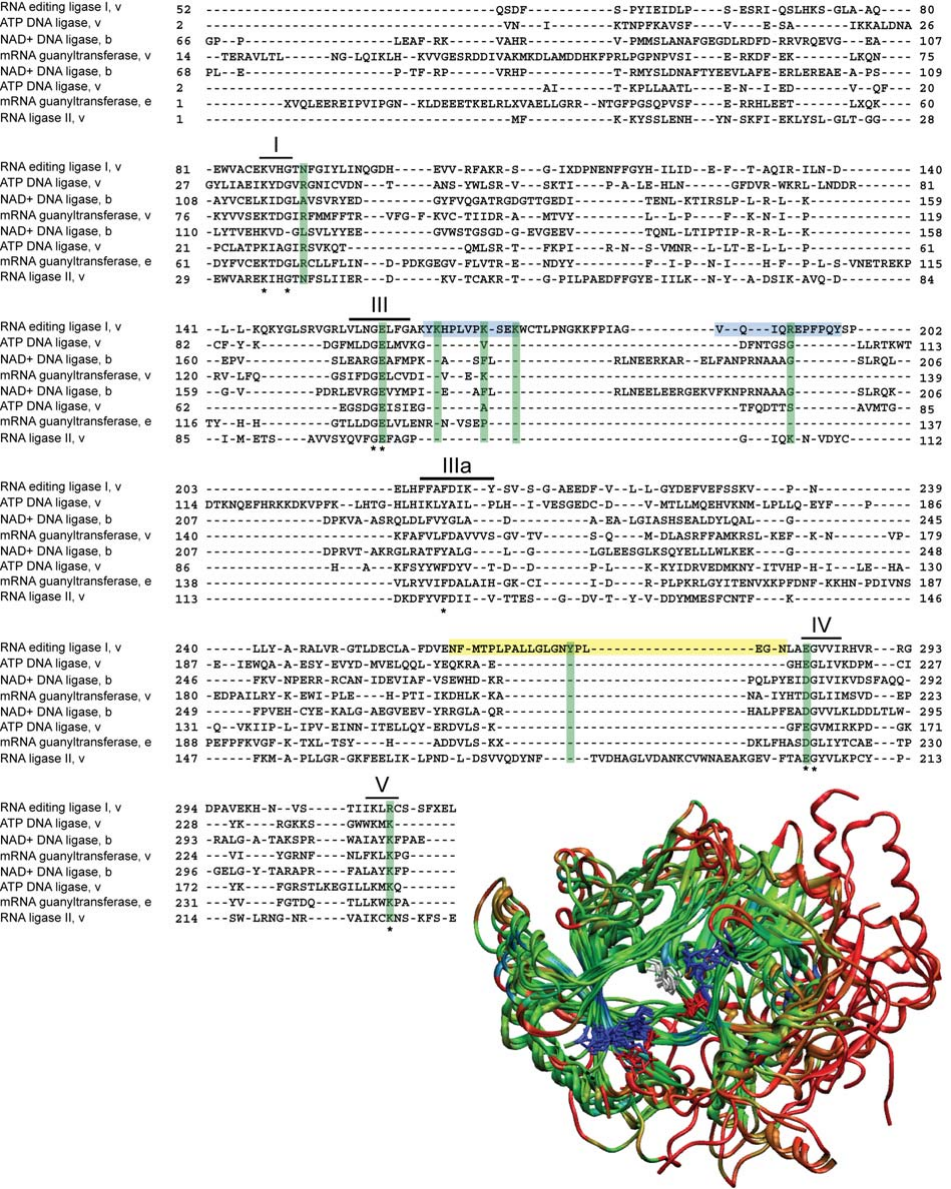
Structural alignment of the covalent nucleotidyl transferase superfamily. Structural alignment of the superfamily of enzymes reveals 8 highly conserved residues (marked with asterisks). The enzyme name, domain of life (v–virus, e–eukarya, b–bacteria), and numbering is provided for each member. The five conserved motifs from the RNA ligase family are indicated with lines and roman numerals. The loops predicted to be involved in RNA binding and interdomain signaling are highlighted in blue and yellow, respectively, and the residues suggested for mutation are highlighted in green. The structurally aligned proteins are shown, colored by conservation, with blue and green indicating more conserved regions and red indicating the most variable regions. The eight conserved residues identified in the structural alignment are depicted in licorice and colored by residue type (hydrophobic in white, negatively charge in red, positively charged in blue).

TbREL1 belongs to the covalent nucleotidyl transferase superfamily of enzymes, along with other RNA ligases, mRNA capping enzymes, and DNA ligases [16]. A thorough bioinformatics and phylogenetic analysis of the RNA ligase family shows five well-conserved structural motifs responsible for the three-step nucleic acid repair and strand-joining reaction, a shared overall protein fold, and common evolutionary traces. At the level of the superfamily, the percent identity among the sequences is less than 10%, which renders traditional sequence alignment measures ineffective. However, a structural alignment of an unbiased and nonredundant set of the members of the superfamily, which includes RNA ligase II, mRNA capping enzyme, NAD+ dependent DNA ligase and the ATP dependent DNA ligase, indicates that there is a well-conserved core structure surrounding the nucleotide binding site (Fig. 2). Furthermore, this analysis reveals eight highly conserved residues that may play key catalytic roles. The structural differences within the nucleotide binding sites are likely due to the subtle differences in their cofactor specificities. A structural phylogenetic analysis based on the multiple structural alignment of the superfamily indicates that the closest known relative to TbREL1 is T4 phage RNA ligase 2 (Fig. S1), and this is consistent with a previous sequence-based evolutionary analysis of the family [16]. Steady-state and pre-steady-state kinetic analysis coupled with strategic mutagenesis of the T4 RNA ligase 2 has established functional roles for the highly conserved binding site residues and mapped many of the important interactions of the RNA ligase active site [17],[18]. The established similarity between these two enzymes is important, as much of this information for the T4 phage system can be used to help interpret, understand, and direct strategic studies for the enzymatic activity of TbREL1.

The existence of a high-resolution ligand-bound crystal structure and unique active site features, coupled with the fact that there is no close human homolog, make TbREL1 an important target for development of inhibitors against these protozoan parasites. In this work, we use all-atom explicit solvent molecular dynamics (MD) simulations to probe the structure, function, and dynamics of TbREL1 on the nanosecond timescale. A comprehensive structural and sequence alignment of all the known superfamily members identifies several key residues that we monitor throughout the simulations. Deeply buried water molecules within the nucleotide binding site and their effect on the mode of ATP-binding are also investigated. A comparison of the principal components for the apo and ATP-bound systems illustrates large local and global differences in the enzyme motion. Ensemble-averaged electrostatics calculations from the MD simulations reveal a putative RNA binding site near the ligase active site, a finding that has previously eluded scientists. Coupled with the crystal structure and currently available experimental information, the dynamics and structural analysis presented here will likely prove to be salient aspects of future successful drug design efforts against this vital enzyme.

## Materials and Methods

### Molecular Dynamics Simulations

Structural coordinates were taken from PDB 1XDN [14]. TbREL1 has a fifty residue N-terminal segment that is believed to be a mitochondrial import signal, which was cleaved before crystallization. The simulated protein contains residues 52–365 of the 469 total residues, thus representing the N-terminal domain. All crystallographically resolved water molecules were retained in the simulations.

In order to prepare the apo-system, the ATP and single Mg^2+^ ion were removed from the active site and replaced by 6 waters using the program Dowser [19]. The selenomethionines used for crystal structure refinement were replaced with methionines. Histidine protonation states were determined using the WHATIF program and manually double-checked. All other hydrogens were added according to the Charmm27 topology parameters [20] using PSFGEN within NAMD2.5 [21]. The protein was immersed in a rectangular box of TIP3P waters [22] providing a 10 Å buffer from the protein to the periodic boundary in each direction. Four sodium atoms were randomly placed at least 5 Å away from the protein in order to neutralize the system's charge. The apo system is comprised of a total of 35,464 atoms.

In order to remove spurious contacts, a set of 26,000 energy minimization steps were carried out. The first 6,000 steps were performed in three 2,000 step cycles. Hydrogen was relaxed during the first 2000 steps, holding all other atoms fixed. Hydrogen, water and ions were relaxed during the next 2000 steps. In the last cycle, the protein backbone was fixed, minimizing all other atoms. No constraints were applied during the last 20,000 steps, freely minimizing all atoms.

Molecular dynamics simulations were carried out for twenty nanoseconds with no constraints and a 1 fs timestep at 1 atm pressure and a temperature of 298.15 K. The temperature bath was maintained by Langevin dynamics while pressure was maintained with the hybrid Nose Hoover - Langevin piston method [23], using period and decay times of 100 and 50 fs, respectively. The Particle Mesh Ewald algorithm was used to treat long-range electrostatics without a cutoff [24]. A multiple time-stepping algorithm was employed, where bonded interactions were computed at every time step, short-range non-bonded interactions were computed every 2 time steps, and full electrostatics were computed every 4 time steps. All minimization and molecular dynamics were carried out using NAMD 2.5. Simulations were performed on our own local cluster, as well as the San Diego Supercomputer Center's Datastar machine and the National Center for Supercomputing Applications' Cobalt machine. A typical benchmark for the 35,000 atom system on 64 processors on NCSA's Altix platform is 0.12 days per nanosecond of simulation. System configurations were sampled every 500 fs, generating 40,000 coordinate snapshots for subsequent analysis.

The ligand-bound system, with ATP and the single magnesium ion present, was prepared in an identical fashion, except that the crystallized ATP and Mg^2+ ^ion were included. An additional 20 ns simulation was performed on the ligand-bound system with identical simulation parameters as described above.

Trajectory analysis was performed with VMD, Matlab, and customized scripts. Ensemble averaged electrostatics calculations were performed in VMD with the PME Electrostatics Plugin. Snapshots every 5 ps over the course of the 20 ns simulation (4000 snapshots) were used in the calculations. All images were created using VMD.

## Results and Discussion

### Comparative Dynamics of the Apo and ATP-bound Systems

**Figure 3:**
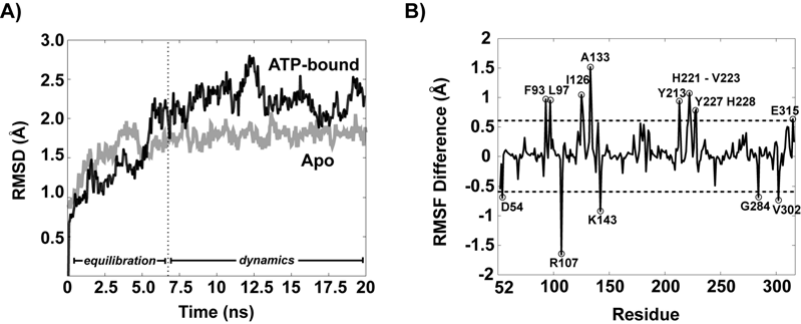
RMSD and RMSF difference analysis for apo and ATP-bound systems. A) The overall RMSD of the protein backbone over the 20 ns simulation is shown for the apo (grey) and ATP-bound (black) systems. B) The average C_α _RMSF per residue over the dynamics phase for all residues in the apo system is subtracted from the average RMSF per residue for the ATP-bound system, illustrating the relative effect that the presence of ATP has on the amount of fluctuation variance exhibited per residue.

Two 20 nanosecond simulations were carried out on the apo and ATP-bound TbREL1 systems in order to investigate their stability and dynamical properties. Both systems were simulated without constraints and reached equilibrium after approximately 7.0 ns of constant pressure and temperature equilibration at 1 atm and 298 K. Correspondingly, the trajectory time from 0–7 ns is referred to as the “equilibration phase” and from 7–20 ns is referred to as the “dynamics phase” (Fig 3).

Plots of the time evolution of the root-mean-square-deviation (RMSD), where each trajectory frame was aligned to the initial starting structure in order to remove any rotational or translational motion, indicate that equilibration was achieved for both systems (Fig. 3A). The overall root-mean-square-fluctuations (RMSF) per residue was calculated during the dynamics phase for each system based on C_α_ positions after alignment to the average equilibrated structure, and the resulting RMSF difference plot shows the differences in RMS fluctuations between the two systems (Fig. 3B). Our results indicate that the presence of ATP appreciably affects the system dynamics, providing stabilization to some regions of the protein structure, while causing increased fluctuations in other areas, relative to the apo-system. Notably, the peripheral loop comprised of residues 213 to 223 exhibits marked increases in flexibility when ATP is bound. The residues with the highest destabilization upon ATP binding are found in the middle of helix-2, A133 and Q134. The increase in fluctuation is due to a significant sidechain and backbone conformational transition of Q134 at 7 ns that allows the amine group of Q134 to change hydrogen bonding partners from the backbone carbonyl of N239 to that of E130. Not surprisingly, the RMSF of adjacent A133 is also affected. Among the most stabilized residues are R107 and D54. The formation of a salt bridge between R153 and D54 at 7 ns prevents excessive motion of the D54 sidechain, which would otherwise make hydrogen bonds to solvent water molecules.

#### Motif Motion

To investigate the differences in local and global motion between the apo and ligand-bound systems, mean RMSF values over each of the five conserved motifs (or residues subsets within each motif) flanking the ATP binding site are reported in Table 1. Motifs I and III exhibit nearly the same RMSF fluctuations for both the apo and ATP-bound states, whereas motifs IIIa and IV are stabilized by ATP and motif V shows increased fluctuations.

**Table 1:** Comparative dynamics of the five conserved motifs and the conserved peripheral loop.

Motif	Residues	Apo RMSF	ATP-bound RMSF
I	K87–G90	0.52	0.53
III	V155–G162	0.79	0.75
IIIa	207–F209	0.76	0.62
IV	E283–I287	0.85	0.74
V	I305–R309	0.42	0.53
	F262–A282	1.58	2.11

Mean RMSF (in angstroms) over the dynamics phase (7–20 ns) of residues within motifs I-V and the conserved peripheral loop, for the apo and ATP-bound system.

**Figure 4:**
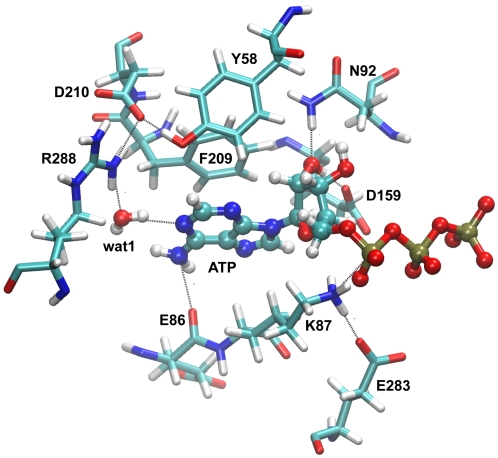
Protein-ATP interactions. Interactions between the single water molecule, protein, and ATP, which persist throughout the ATP-bound simulation, are shown.

**Figure 5:**
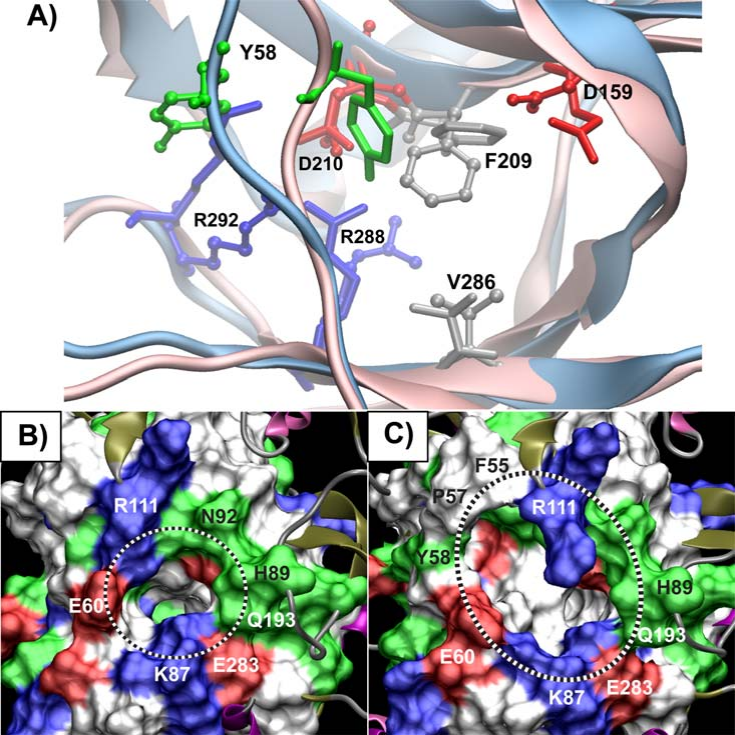
ATP binding pocket undergoes significant rearrangement in the apo state simulations. In all panels, residues are colored by residue type: red–acidic, blue–basic, white or gray–hydrophobic, green–polar. A) A structural alignment of a representative structure from the apo simulation (backbone shown in blue cartoon, residues shown in ball and stick) and the ATP-bound simulation (pink cartoon, residues shown stick-only licorice) shows the relative reorientation of key active site residues. B) MSMS surface representation [30] of residues within a 12 Å radius of the ATP binding pocket at the beginning of the apo trajectory. Residues on the periphery are labeled, and the ATP binding pocket is highlighted with a dotted line. C) Same as in (B), but after 20 ns of simulation (structure is same as the blue structure in panel (A)). An additional cavity opens between R111 and E60, exposing several new polar and hydrophobic residues.

Motif IIIa is one of two motifs exhibiting less motion in the ATP-bound system (Table 1). This decrease in RMSD is associated with two stabilizing interactions in motif IIIa: the D210-R288 salt bridge and a pi-stacking interaction between F209 and the adenine ring of ATP (Fig. 4). Throughout the 20 ns ATP-bound system simulation, both of these contacts remain intact (Tables 2 and 3). Conversely, in the apo system simulation, both of these stabilizing contacts are disrupted (Table 3). The D210-R288 salt bridge is disrupted at 5 ns, when D210 forms a salt bridge with nearby R292. Furthermore, in the apo simulation, with no ligand present to stabilize it, F209 undergoes a large swinging motion, eventually resulting in a rearrangement of the active site cavity (Fig. 5A).

**Table 2:** ATP-TbREL1 interaction analysis.

Motif	Protein	ATP	Mean (Std Dev)	Crystal
I	K87:NZ	PA	4.04 (0.09)	3.66
III	E159:OE	O2'	2.63 (0.12)	2.66
IIIa	F209	Adenosine	3.87 (0.23)	3.77
V	R309:NH2	O1B	3.65 (0.65)	2.98
V	K307:NZ	O2A	2.70 (0.15)	3.52
	E86:O	N6	2.88 (0.16)	2.91
	R111:NH2	O3G	4.93(1.03)	2.81
	N92:ND2	O2'	3.09 (0.26)	2.90

The interactions between conserved residues in TbREL1 and ATP are reported for the 20 ns simulation (Fig. 4 for structural depiction). For the F209–Adenosine pi-stacking interaction, the distance between the centroid of each of the rings is reported. The mean over the simulation and the standard deviation (in parentheses) are reported, as well as the distance measured in the crystal structure.

**Table 3:** Protein interaction analysis.

Protein	Protein	Apo Mean (Std Dev)	ATP-bound Mean (Std Dev)	Crystal
D210:OD2	R288:NH2	5.52 (0.94)	3.22 (0.35)	3.56
D210:OD2	R292:NH1	3.10 (0.45)	3.75 (0.72)	2.65
Y58:OH	D210:OD2*	5.56 (2.49)	3.05 (0.75)	3.95
Y58:OH	R288:NH1	10.00 (2.52)	3.78 (0.30)	4.14

Interaction distances are reported between the atoms specified in the first two columns (see Fig. 4 for structural depiction). The mean interaction distance and standard deviation is determined over the 20 ns simulation. The final column is the interaction distance reported in the crystal structure.

Motif IV is also stabilized in the ATP-bound system. Significant stabilization of G283 and E284 is the result of an electrostatic network between E284, nearby charged residues K87 and R309, and the polyphosphate tail of ATP.

Motif V is the only motif that is significantly destabilized in the ATP-bound system. The motif is destabilized as L308 and R309 undergo significant C alpha, beta, and gamma rearrangements as R309 twists about its C_β_, C_γ_, and C_δ_ bonds to optimize its electrostatic interactions with neighboring E284 and the polyphosphate tail of ATP. The motion of R309 is mainly constrained to its alkyl chain and not its charged end, which is pinned between E284 and the β-phosphate group of ATP (Table 2).

### TbREL1-ATP Interaction Analysis

An analysis of the interactions between the bound ATP and conserved TbREL1 residues over the course of the MD simulation can be used to gain insight into the complex dynamics, as well as to predict solution state behavior of the enzyme-substrate complex. Here we describe the conserved ATP-protein interactions, which can be broadly categorized into three groups based on their proximity to the ATP adenosine moiety, ribose, or triphosphate tail.

The adenosine moiety of ATP sits deep within the binding site; residues that interact with the adenosine moiety include E86 and F209. The carbonyl oxygen of E86 accepts a hydrogen bond from ATP's amine and helps lock ATP into position, preventing it from translating fore and aft in the binding pocket. The phenyl group of F209 provides important stabilizing pi-pi interactions with the adenosine moiety, further attenuating lateral movement of ATP within the active site. Both the F209 and the E86 interactions have relatively low standard deviations and average values close to those observed in the crystal structure (Table 2) demonstrating that these interactions are among the most stable in the protein-substrate complex.

Two conserved residues interacting with the ribose moiety are E159, N92. This pair of residues forms a unique hydrogen bonding pattern with the ribose O2' that was conserved throughout the 20 ns simulation (Table 2). The ribose O2' hydroxyl group donates a hydrogen bond to the carboxyl group of E159, while the amine group of N92 donates a hydrogen bond to the ribose O2' (Fig. 4). The slight deviation between the bond distances observed in the crystal structure and the average values observed during the simulation is attributable to conformational fluctuations within the ribose ring from the slightly more compact C-3 and C-2 endo conformations to the more extended envelope conformation.

A number of charged residues near the periphery of the binding site, including R309, K307, K87, R111, interact with the polyphosphate of ATP. K307 and R111 primarily stabilize the conserved triphosphate conformation observed over the course of the 20 ns simulation. The interaction distance between K307 and the O2A of ATP is 0.82 Å shorter than that observed in the crystal structure. This is attributable to rotation about the O5'-P_α _bond that brings the O2A closer to the K307 amine. The low standard deviation exhibited throughout the trajectory illustrates the importance of this interaction in maintaining the triphosphate conformation (Table 2).

While it is not immediately apparent from the 4.9 Å average R111 NH–O3G interaction distance, R111 also contributes important triphosphate longer-range stabilizing interactions. For the first 1.2 ns, R111 samples conformations local to that found in the crystal structure, with the R111 NH2 group proximal to O3G. Subsequently, R111 rearranges by rotating through the CD-NE bond whereby the R111 NH1 group donates a hydrogen bond to triphosphates bridging O2B while the NH2 group maintains a hydrogen bond with O3G. Following this rearrangement, a rotation about the NE-CZ bond occurs, which exchanges NH1 and NH2 hydrogen bonding partners while preserving the hydrogen bonding pattern. This second rotation moves the NH2 group 5.2 Å from O3G, and because it occurs frequently, is responsible for the high standard deviation and longer average interaction distance (Table 2). The K87 electrophile remains at an unreactive 4.0 Å average from the alpha phosphate nucleophile. This agrees with crystal structure data from other superfamily members, which suggest that the triphosphate tail must undergo a conformational rearrangement to properly position the alpha phosphate for catalysis [25]. Because of its relatively distal position and larger standard deviation, we predict R309 plays a secondary role in triphosphate stabilization.

### Dynamics of Conserved Residues within the Binding Site

The interactions of several conserved residues within the ATP binding site exhibit altered dynamics in the apo and ATP-bound systems (Table 3). Here we provide insight into the stability of the protein complex by examining how these protein interactions are affected by the presence of ATP in the binding pocket.

In the ATP-bound state, D210, R288 and Y58 form a tight hydrogen bonding and electrostatic network at the deep end of the active site (Fig. 4). D210 interacts with R292, but because it is already involved in a stable network of three hydrogen bonds, it makes a poor binding partner. As a result R292 undergoes large fluctuations relative to those observed in the D210, R288, Y58 triad (Table 3). A significantly different behavior is seen in the absence of ATP. In the absence of the pi-pi stacking interactions provided by the adenosine moiety, F209 swings inward toward the active site cavity, disrupting the D210, R288, Y58 triad. Once the network of interactions is destabilized, thermal fluctuations cause R288 to swing away from D210 causing a 1.96 Å increase in average bond distance relative to that observed in the crystal structure (Table 3). D210 subsequently forms a salt bridge with R292 whose average is 0.83 Å shorter than that observed in the crystal structure. This new interaction pattern is conserved throughout more significant active site rearrangements that were observed later in the simulations, as discussed in greater detail below.

### ATP-binding Induces Fluctuations at Remote Sites

TbREL1 has several important loop regions on the periphery of the ATP binding site that may be functionally relevant. One region in particular, a helix-loop segment formed by residues F262-A282, is highly conserved among all trypanosomatids and has been hypothesized to be involved in protein-protein association within the editosome complex (Fig. 2) [14]. Comparing the backbone RMSF values for the apo and ATP-bound system reveals that ATP binding induces a significant increase in motion within this conserved helix-loop region (Table 1). As this region is not directly linked to the ATP binding site, the propagation of this motion must be through cooperative interactions. This is further supported by a principal components analysis (Supplementary Information), which shows that motion in this helix-loop region is primarily accounted for in the most dominant principal components for the ATP bound system, and that the motion is not present in the apo system (Fig. S2). These results suggest that these loop regions may play an important role in interdomain signaling or crosstalk upon ATP binding.

### Active Site Rearrangement in Apo System

During the apo system simulation, a significant rearrangement of the active site cavity is observed. In the absence of ATP, the active site pocket is initially solvated with water molecules. After 6.5 ns, F209 swings out of the binding pocket towards Y58, making favorable hydrophobic contacts with the sidechain of I305. This structural reorganization forms a hydrophobic barrier to the deep end of the active site, occluding penetration of solvent water molecules (Fig. 5, Dataset S1).

A radius of gyration analysis for motifs IIIa and IV indicates that in the absence of ATP the formation of the hydrophobic barrier leads to a slight contraction of the distance between motifs IIIa and IV that comprise the deep end of the binding pocket (Fig. S3). Further rearrangement occurs after 12.5 ns when Y58 swings away from motif IIIa, towards the bulk solvent. Over the course of the simulation, the distance between motif IIIa and the strand housing residues 58 to 61 systematically increases from 7 Å initially to almost 13 Å, as measured by the distance between the alpha carbons of Y58 and R111.

These results suggest a strong induced fit effect may occur upon ATP binding. The structural reorganization and increased overall flexibility of TbREL1 without ATP-bound (Table 3) may explain the difficulty in crystallizing the apo structure experimentally (personal communication, J. Deng). In addition, the rearrangements observed in the simulations reveal an altered topography near the ATP binding site, which could potentially be exploited in a structure-based inhibitor design scheme in which compounds could be designed that stabilize this inactive conformation. The strategy of targeting a unique inactive conformation was successfully accomplished with Abelson tyrosine kinase and the inhibitory compound Gleevac, which locks the kinase in an inactive conformation with high specificity and affinity [26],[27]. To expedite and assist drug discovery efforts against TbREL1, we are providing a representative reorganized apo structure extracted from the simulations as Dataset S1.

### Role of Water Molecules in the Binding Site

The TbREL1 crystal structure revealed three deeply buried water molecules coordinating interactions between conserved active site residues and ATP [14]. The simulations presented here include all the crystallized water molecules, allowing us to monitor their behavior over the course of the 20 ns trajectory. For continuity between the crystal structure and this work, we adopt the water molecule naming convention as defined in Ref. Deng et al, 2004. These deeply buried water molecules are a unique feature of TbREL1, therefore it may be advantageous to consider them in the inhibitor design process. In particular, insights into their exchange rates and structural features are of interest.

In the ATP-bound system, wat1, which forms bridging hydrogen bonds between conserved R288 and the N1-adenine group of ATP (Fig. 4), remains fairly rigid in its position throughout the equilibration phase. Wat2, which initially forms a hydrogen bond with the backbone carbonyl of conserved V286, immediately changes position to form a hydrogen bond network with wat1, R288 and D210. Once this initial rearrangement occurs, the new configuration remains for the duration of the equilibration phase.

During the dynamics phase of the ATP-bound system, two of the three deeply buried water molecules exchange with bulk solvent. Wat3, which is initially coordinated to wat 1, the backbone carbonyl of F209, and the charged group of R288, is the first of the buried water molecules to exchange. Wat1, the single water molecule that interacts directly with the adenine ring of ATP, is the only buried water molecule that does not exchange. The exchange of these water molecules and subsequent rearrangement of conserved active site residues may indicate that they help modulate the plasticity of the ATP binding site and that it may be possible to advantageously displace one or more of these water molecules with inhibitor functional groups. Furthermore, during the dynamics phase of the ATP-bound system, additional water molecules gain access to the ATP-binding pocket and lubricate interactions between the ligand and the protein. These additional water molecules contribute to the expansion of the active site cavity as seen in the radius of gyration analysis (Fig. S3).

### Ensemble Averaged Electrostatics Calculations

To date, the RNA binding region of TbREL1 has eluded scientists. Despite the high resolution crystal structure, a surface electrostatics calculation performed with DelPhi on the crystal structure did not reveal any large positive patches on the surface of the enzyme where RNA binding might occur [14].

**Figure 6:**
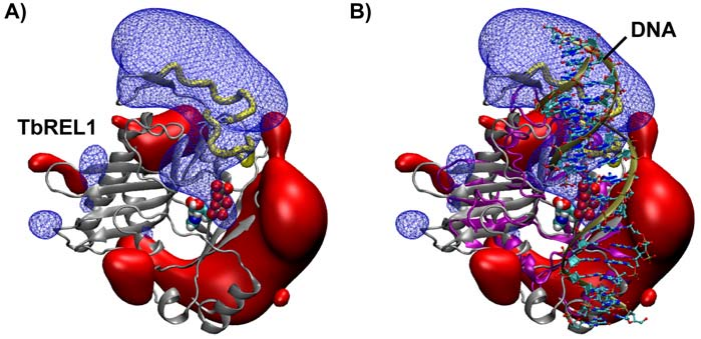
Ensemble averaged electrostatics for TbREL1 predict RNA binding site. Negative electrostatic isosurface is shown in red solid, positive isosurface in blue wireframe; isosurfaces are drawn at −70 e/kT and +70 e/kT. A) TbREL1 is shown in silver cartoon with ATP-bound in the active site (depicted in space-filling). Two unique loops in TbREL1 are shown in yellow tube. B) TbREL1 superimposed with the crystal structure of the homologous human DNA ligase (depicted in purple cartoon) shows that the positive electrostatic lobes of TbREL1 overlap well with the location of DNA co-crystallized with human DNA ligase.

To further investigate the electrostatics of TbREL1, we calculated the electrostatic potential around the ATP-bound protein using ensemble averaging over the 20 ns trajectory. This analysis reveals a large positive electrostatic lobe near the ligase active site, clearly indicating where RNA could favorably bind (Fig. 6A). Furthermore, a structural alignment of TbREL1 with the related human DNA ligase shows nearly perfect overlap of its DNA-binding region with the putative RNA binding region predicted here (Fig. 5B).

Within the center of the putative RNA binding site are two peripheral loops that are unique to the trypanosomes (Y165 - K175 and V190–Y200). These loops had previously been speculated to be involved in RNA recognition due to the presence of a positively charged residue within each, K166 and R194, respectively [14]. The ensemble averaged electrostatic potential maps presented here provide compelling evidence that these loops may be involved in RNA binding, and they are particularly encouraging in light of a recent study that showed extensive MD simulations can accurately reproduce experimentally observed changes in protein electrostatic fields [28]. Interestingly, these putative RNA binding loops are absent in the N-terminal domain of homologous T4 RNA ligase 2, which, in the absence of its C-terminal domain, is not able to catalyze the ligation reaction [18], unlike TbREL1, which is still active in the isolated N-terminal domain [14]. The large positive electrostatic potentials substantiate the idea that TbREL1 is equipped with its own RNA binding motifs, which allows it to retain activity even in the absence of a C-terminal OB-fold domain.

### Conclusions and Experimental Predictions

The results presented here allow us to make several predictions that may help motivate future experiments on this vital enzyme. As no single experimental or theoretical approach will answer all questions regarding enzyme function, complex association, and its role in the vitality of the cell, we suggest a variety of approaches to probe the function of TbREL1 and its interactions with the multicomponent editosome complex.

The persistent hydrogen bond interactions formed between N92 and E159 and the ribose groups of ATP (Table 2, Fig. 4) indicate that these residues may play an important role in ATP binding. Mutagenesis studies of the equivalent of N92 in homologous T4 phage RNA ligase 2 substantiates this hypothesis [17]. We predict that mutation of either or both of these residues will decrease ATP binding, most likely through an increase of the K_m_ of ATP for this enzyme. As a negative control, we predict that mutation of nonconserved residues far away from the ATP binding pocket should not affect ATP binding, such as N239A or I98A.

The adenylation of TbREL1 is a key component of the overall reaction catalyzed by this enzyme. K87 has been shown to be the adenylated active site residue, as even the charge-conservative mutation K87R abolishes ligation activity [29]. The triphosphate tail was crystallized in an unreactive conformation relative to K87 that persists throughout the duration of the trajectory (Table 2), which suggests that the second magnesium, the C-terminal domain, RNA substrate, or some other component of the editosome complex needs to bind before the TbREL1 active site will adopt a catalytic conformation. Repositioning of the polyphosphate tail is necessary in order to bring the alpha-phosphate proximal to the K87 electrophile and in-line for attack. This is also suggested by previously reported structural data on other superfamily members [25]. We predict that nearby R309 plays a secondary role in triphosphate stabilization and that mutation of this residue will reduce the rate of catalysis but not abolish it. The interactions between the non-bridging beta and gamma triphosphate oxygens and the charged end of R309 may help stabilize the polyphosphate tail for the in-line attack. Furthermore, E283, one of only eight residues conserved in both structure and sequence for the entire superfamily, is likely the catalytic base in the ligation reaction. It is the only negatively charged residue within reasonable proximity of the catalytic K87, and it remains oriented in a position that would promote catalysis throughout the simulations (Table 2, Fig. 4). This is again substantiated by mutagenesis studies of the equivalent conserved glutamic acid residue in the closely related T4 phage RNA ligase 2 [17].

Based on the RMSF (Table 1) and principal components analysis (Fig. S2), we predict that the conserved peripheral loop comprised of residues F262-A282 may play an important role in interdomain signaling upon ATP binding. It may be possible to test this by engineering a fluorescent probe (e.g. Y275W) in this loop region and then testing whether the fluorescence signal is quenched with the addition of various components of the editosome complex (TUTase, KREPA2, etc), which would indicate that the region is buried and in contact with another editosome protein.

Our simulations and analysis also suggest that the pi-stacking interactions between conserved F209 and the adenosine ring of ATP are of critical importance to stabilizing TbREL1 tertiary structure. Without the ring stacking interactions provided by ATP, the apo protein was greatly destabilized in the vicinity of F209 (Fig. 5). We predict that inhibitors that are competitive with ATP will need to exploit this moiety, providing stabilizing interactions to the core protein structure, either through similar pi-stacking interactions or general hydrophobic interactions. In addition, the exchange of water molecules at the deep end of the ATP binding pocket may indicate that they could be replaced with inhibitor functional groups.

Ensemble averaged electrostatics calculations have allowed us to predict the putative RNA binding region for TbREL1 (Fig. 6). Two unique loops, comprised of residues Y165 - K175 and V190–Y200, are predicted to be near the center of the RNA binding region. We propose that mutation of the positively charged groups in this region, such as K166, K172, K175, or R194, to either neutral groups or negatively charged groups, should alter the electrostatic potential, disrupt the rate of association with the RNA, and reduce the overall rate of catalysis. After the adenylation reaction takes place, the dissociation of the negatively charged pyrophosphate would further increase the positive potential generated in this region.

RNA editing ligase 1 from *T. brucei* is a promising drug target against African sleeping sickness, and it shares close homology to proteins from the parasites responsible for both Chagas' disease and Leishmaniases, which further underscores its importance to global health. It also presents an ideal system to deepen our understanding of the fundamental and fascinating process of RNA editing. Advancing the knowledge of enzymes and biochemical pathways unique to the trypanosomes, such as mitochondrial RNA editing, will play an important role in the efforts to bring these diseases out of neglected status and into the limelight.

## Supporting Information

Dataset S1 KREL1 apo structure from MD simulations. The reorganized KREL1 apo structure generated after 20 ns of molecular dynamics in PDB format.
(0.33 MB TXT)

Figure S1 Structural Phylogeny of the Superfamily
(0.15 MB DOC)

Figure S2 Principal Component Analysis of the Apo and ATP-bound systems
(0.16 MB DOC)

Figure S3 Radius of Gyration Analysis (0.33 MB DOC)

Text S1 Supporting Information Text
(0.04 MB DOC)
