# Supplementary figures and images for: Functional and Structural Insights Revealed by Molecular Dynamics Simulations of an Essential RNA Editing Ligase in Trypanosoma brucei
Source: PLoS Negl Trop Dis. 2007 Nov 14;1(2):e68. doi: 10.1371/journal.pntd.0000068 (PMC2100368; doi:10.1371/journal.pntd.0000068)

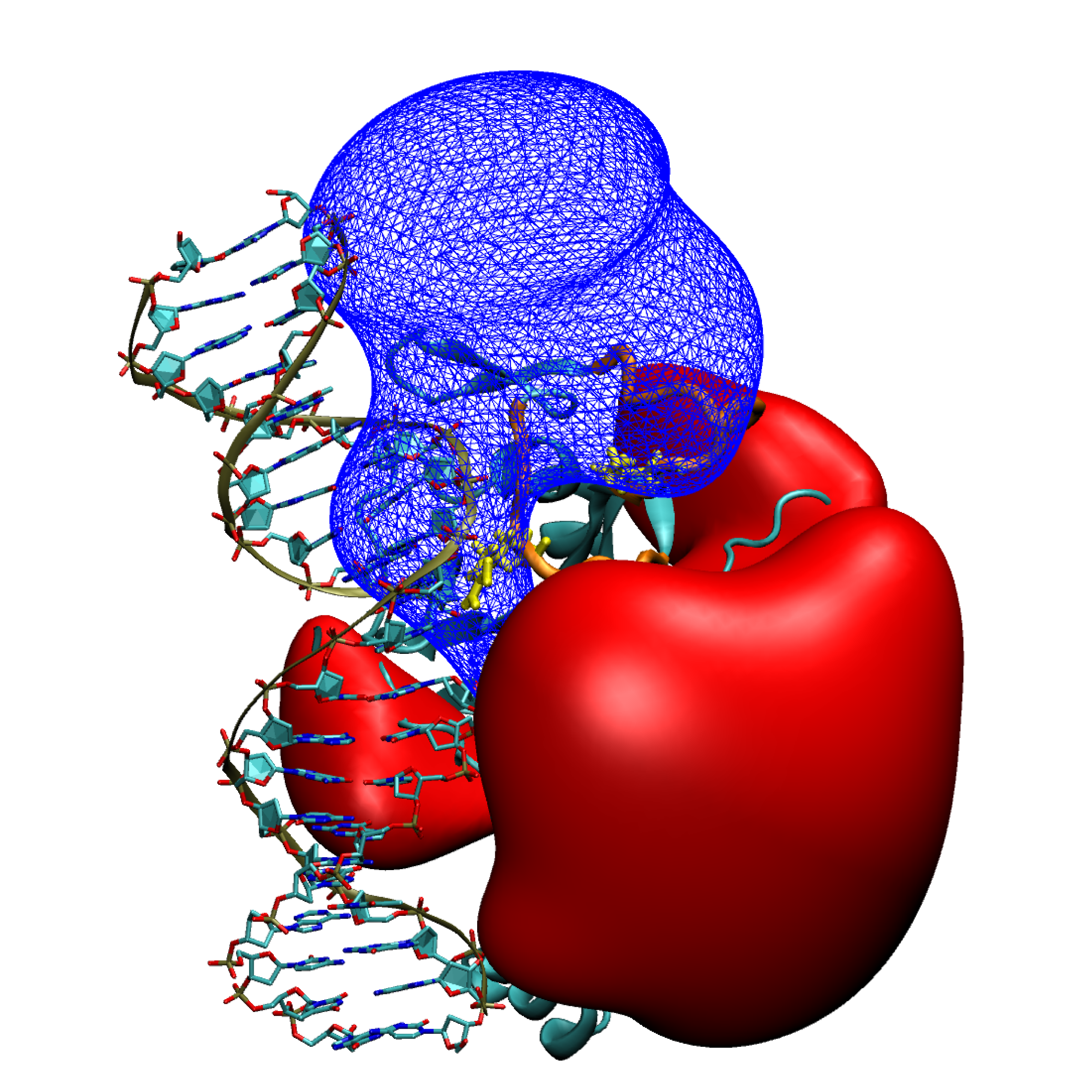

Supplement: Dataset S1 — KREL1 apo structure from MD simulations. The reorganized KREL1 apo structure generated after 20 ns of molecular dynamics in PDB format. (0.33 MB TXT) [file pntd.0000068.s001.txt]
