# Supplementary material for: Functional and Structural Insights Revealed by Molecular Dynamics Simulations of an Essential RNA Editing Ligase in Trypanosoma brucei
Source: PLoS Negl Trop Dis. 2007 Nov 14;1(2):e68. doi: 10.1371/journal.pntd.0000068 (PMC2100368; doi:10.1371/journal.pntd.0000068)
Supplement: Figure S1 — Structural Phylogeny of the Superfamily (0.15 MB DOC) [file pntd.0000068.s002.doc]

**
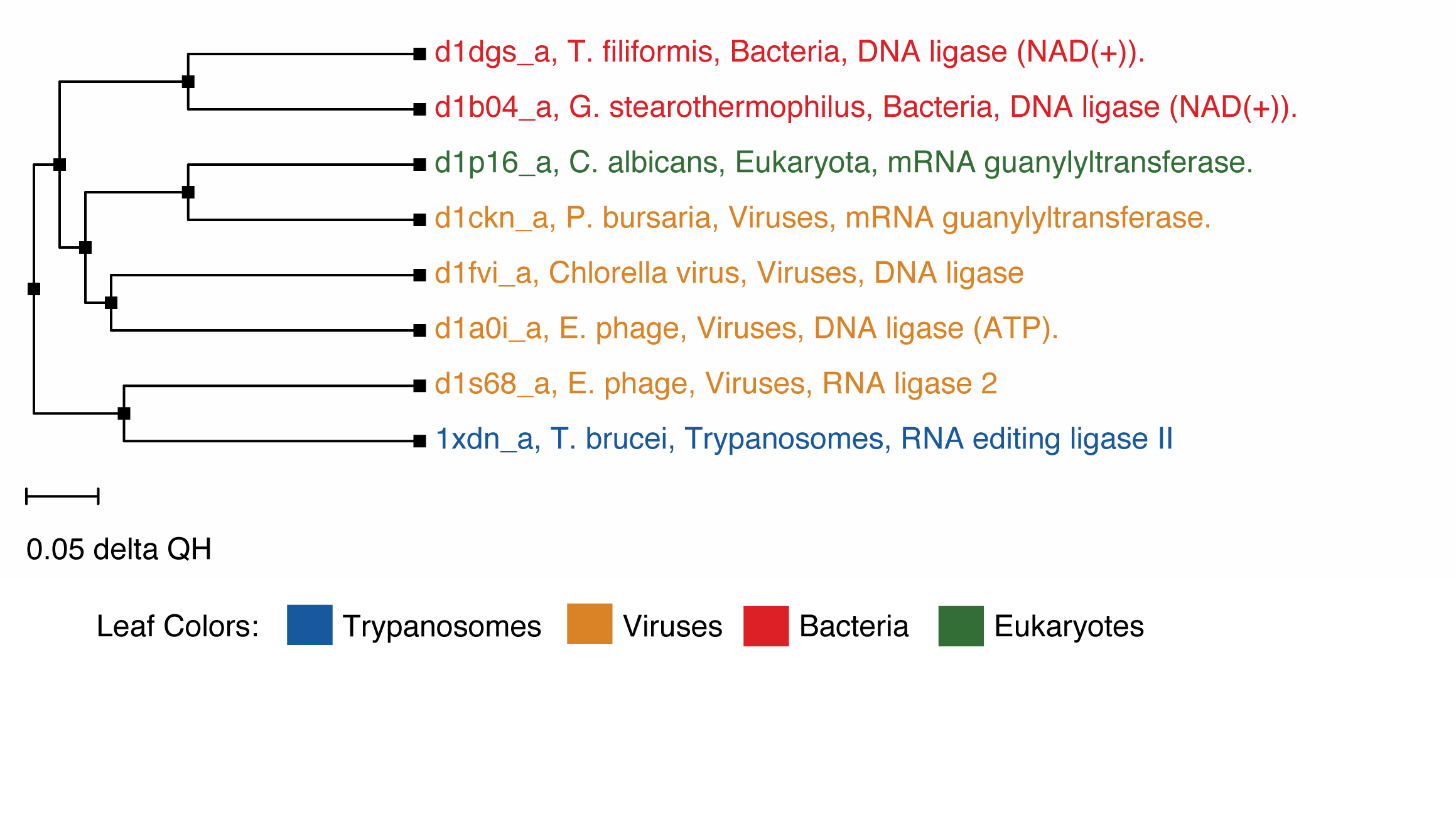
**

**Figure S1: Structural phylogeny of the superfamily. A phylogenetic tree based on the multiple structural alignment performed on all covalent nucleotidyl superfamily members (alignment shown in manuscript Figure 2) is shown above and colored by domain of life. The distance metric used is QH­ and the analysis was performed with the MultiSeq plugin in VMD. Detailed information on the methods used to create the structural phylogenies and their interpretation can be found in Refs. (O'Donoghue and Luthey-Schulten 2003, 2005).**
