# Supplementary material for: Functional and Structural Insights Revealed by Molecular Dynamics Simulations of an Essential RNA Editing Ligase in Trypanosoma brucei
Source: PLoS Negl Trop Dis. 2007 Nov 14;1(2):e68. doi: 10.1371/journal.pntd.0000068 (PMC2100368; doi:10.1371/journal.pntd.0000068)
Supplement: Figure S2 — Principal Component Analysis of the Apo and ATP-bound systems (0.16 MB DOC) [file pntd.0000068.s003.doc]

**
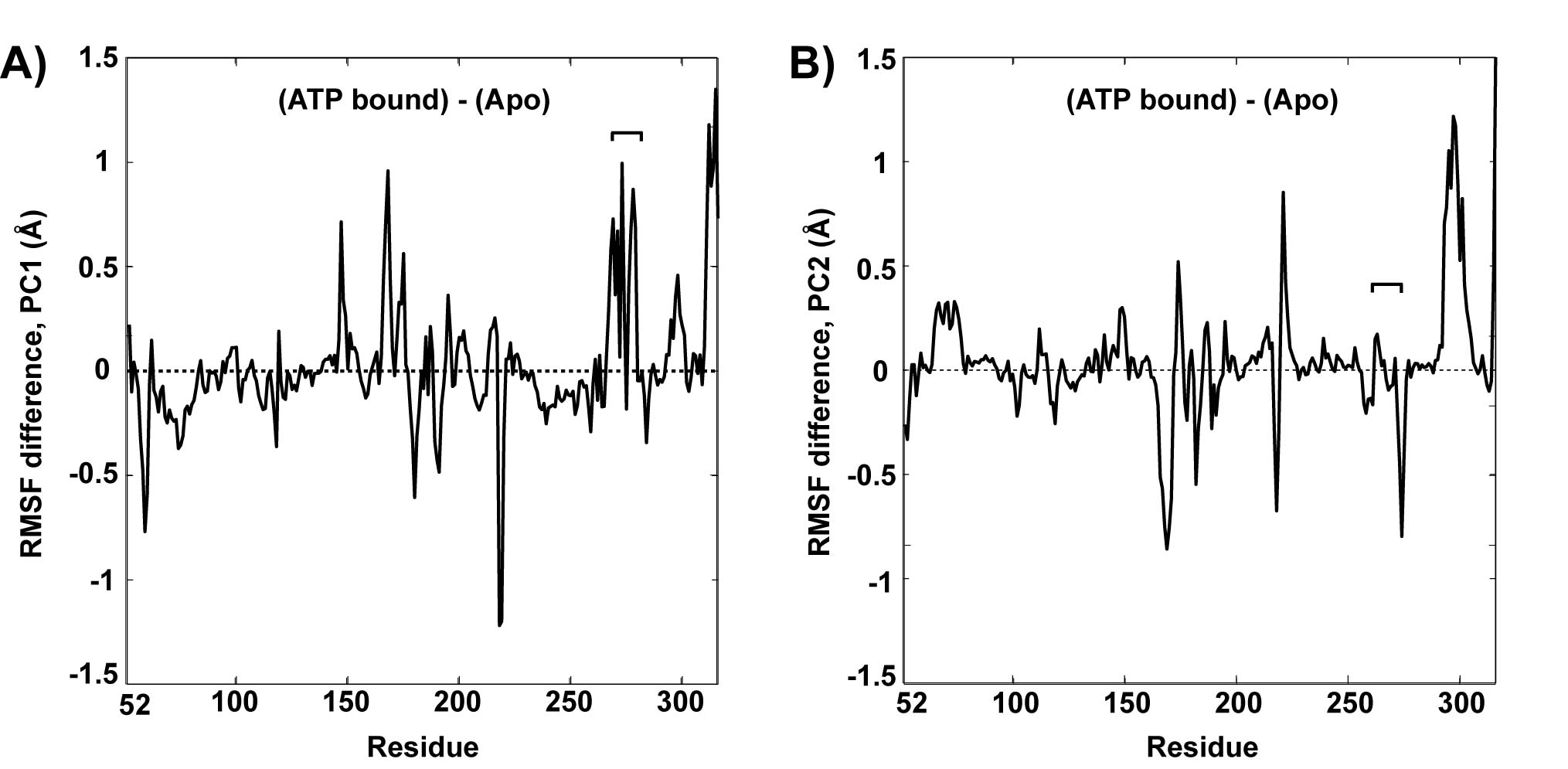
**

Figure S2: Principal Component Analysis of the Apo and ATP-bound systems. A) The RMSF difference per residue due to the first (most dominant) principal component, PC1, indicates the regions of REL1 that are most affected due to the presence of ATP, along the PC1. B) Same as in (A), but for the second most dominant collective motion, PC2. Note that the unique loops predicted to play a role in signaling (I262-A282, denoted with a black bar above the data) exhibit increased fluctuations along the most dominant PC1.
