# Supplementary material for: Functional and Structural Insights Revealed by Molecular Dynamics Simulations of an Essential RNA Editing Ligase in Trypanosoma brucei
Source: PLoS Negl Trop Dis. 2007 Nov 14;1(2):e68. doi: 10.1371/journal.pntd.0000068 (PMC2100368; doi:10.1371/journal.pntd.0000068)
Supplement: Figure S3 — (0.33 MB DOC) [file pntd.0000068.s004.doc]

**
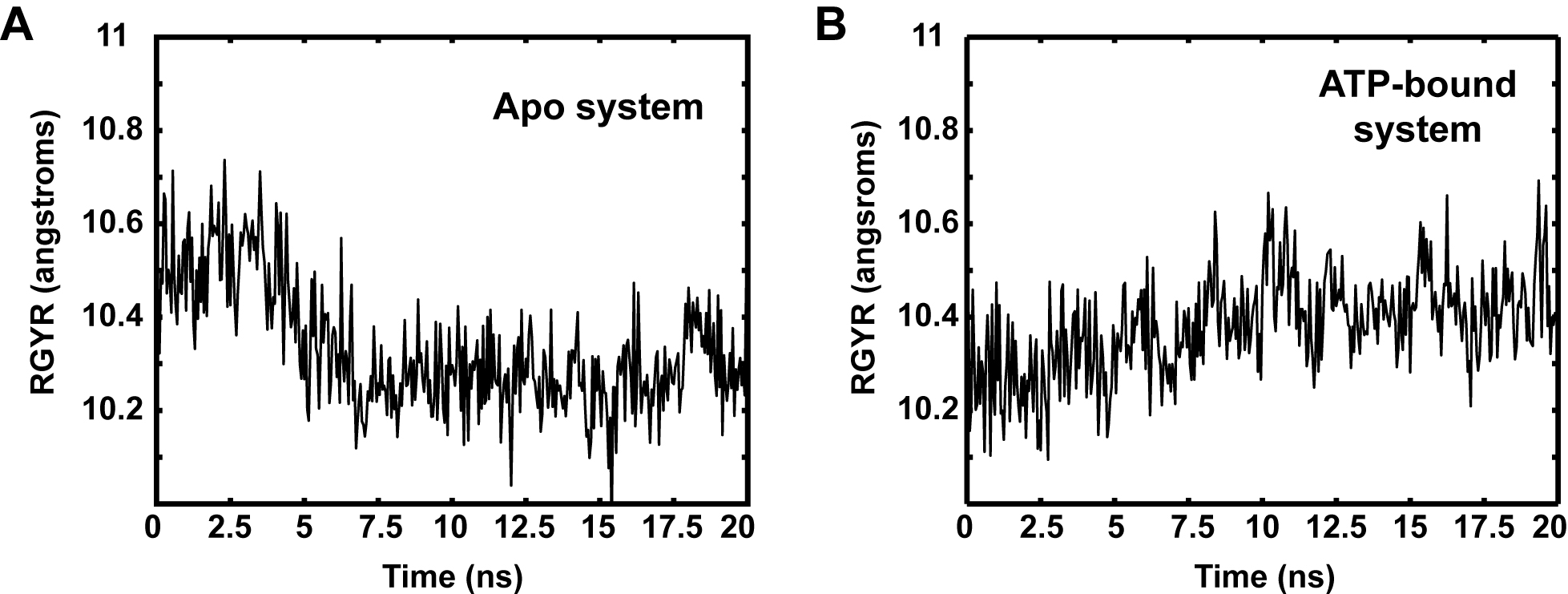
**

Figure S3: Radius of gyration calculated over the 20 ns trajectory for the apo-system (A) and ATP-bound system (B).
