# Supplementary material for: Functional and Structural Insights Revealed by Molecular Dynamics Simulations of an Essential RNA Editing Ligase in Trypanosoma brucei
Source: PLoS Negl Trop Dis. 2007 Nov 14;1(2):e68. doi: 10.1371/journal.pntd.0000068 (PMC2100368; doi:10.1371/journal.pntd.0000068)
Supplement: Text S1 — Supporting Information Text (0.04 MB DOC) [file pntd.0000068.s005.doc]

**Supporting Information**

**Principal Component Analysis**

Principal component analysis (PCA) is used to re-express large data sets in a basis whose components are orthonormal vectors in the direction of maximum data variance (Garcia 1992). By expressing a molecular dynamics trajectory as a linear combination of principal components the covariance, or thermal noise, is eliminated and the underlying collective motions become evident (Hayward et al. 1994; Balsera et al. 1996; Mongan 2004). Principal component analysis was carried out on the C-alpha backbone for the dynamics phase of the trajectory (from 7-20 ns). Translational and rotational degrees of freedom were removed by fitting each configuration to the structure sampled at 500 fs. A zero-mean covariance matrix, *C*, was generated according to equation 1:

(1)

Where the superscript *T* designates the matrix transpose and the matrix *X* is given:

(2)

Where *N* gives the number of residues in the protein, *t* is the total number of configurations sampled and denotes the average over all configurations sampled. The matrix C is diagonalized by determining a set of orthonormal eigenvectors, i.e. the principal components, that satisfy the following equality:

(3)

where *P* is a 3*N* x *3N* matrix whose columns are the principal components and *D* is the diagonalized covariance matrix whose main diagonal contains the corresponding eigenvalues, denoted *λ_i,j_*. That is, *p_i_*, the i^th^ column of *P*, is a vector in a direction with an associated mean-square deviation, or variance, given by *λ_i,i_*. The root mean square fluctuation of residue j, R*MSF_j_*, along principal component *i* was calculated using equation 4:

(4)

where *y_i,j_* is the *j^th^* component of *p_i_*.

**Radius of Gyration**

The radius of gyration (RGYR) was determined using the measure command using the TCL/TK command window within VMD. The formula used to calculate the radius of gyration is given by equation (5):

(5)

Where the sums run over the selected group of atoms, *m(i)* is the mass of atom *i*, *r(i)* is the position of atoms *i* and $\bar{r}$ is the geometric center of the selected group of atoms.

References

Balsera MA, Wriggers W, Oono Y, Schulten K (1996) Principal Component Analysis and Long Time Protein Dynamics. J Phys Chem 100(7): 2567-2572.

Garcia AE (1992) Large-amplitude nonlinear motions in proteins. Physical Review Letters 68(17): 2696-2699.

Hayward S, Kitao A, Go N (1994) Harmonic and anharmonic aspects in the dynamics of BPTI: a normal mode analysis and principal component analysis. Protein Sci 3(6): 936-943.

Mongan J (2004) Interactive essential dynamics. J Comput Aided Mol Des 18(6): 433-436.

O'Donoghue P, Luthey-Schulten Z (2003) On the evolution of structure in aminoacyl-tRNA synthetases. Microbiol Mol Biol Rev 67(4): 550-573.

O'Donoghue P, Luthey-Schulten Z (2005) Evolutionary profiles derived from the QR factorization of multiple structural alignments gives an economy of information. J Mol Biol 346(3): 875-894.
